# Levels of Trace Elements in Erythrocytes as Endocrine Disruptors in Obese and Nonobese Women with Polycystic Ovary Syndrome

**DOI:** 10.3390/ijerph19020976

**Published:** 2022-01-16

**Authors:** Kamila Pokorska-Niewiada, Agnieszka Brodowska, Jacek Brodowski, Małgorzata Szczuko

**Affiliations:** 1 Department of Toxicology, Dairy Technology and Food Storage, West Pomeranian University of Technology in Szczecin, 71-374 Szczecin, Poland; 2 Department of Gynecology, Endocrinology and Gynecological Oncology, Pomeranian Medical University in Szczecin, 71-210 Szczecin, Poland; agnieszka.brodowska@pum.edu.pl; 3 Primary Care Department, Pomeranian Medical University in Szczecin, Żołnierska 48, 71-210 Szczecin, Poland; jacek.brodowski@pum.edu.pl; 4 Department of Human Nutrition and Metabolomics, Pomeranian Medical University in Szczecin, 71-460 Szczecin, Poland

**Keywords:** PCOS, reproductive hormones, trace elements, erythrocytes

## Abstract

Introduction: Polycystic ovary syndrome (PCOS) is one of the most commonly recognized endocrinopathies in women. The literature lacks clear data that allow any meaningful conclusions to be drawn about the influence of trace elements in erythrocytes on the biochemical parameters of PCOS. Materials and methods: This study was conducted among 47 women meeting the Rotterdam criteria for the diagnosis of polycystic ovary syndrome. The research groups included women with PCOS with different BMI values (body mass index): obese women with PCOS (PCOS with BMI ≥ 30, mean BMI index 35.4 ± 4.4 kg/m^2^), nonobese PCOS women (PCOS with BMI < 30, mean BMI index 25.2 ± 2.8 kg/m^2^), and healthy control group (CG) with a mean BMI of 23.57 ± 0.9 kg/m^2^. The contents of trace elements in erythrocytes were determined with an inductively coupled plasma atomic emission spectrometer. Results: The only trace element showing significant differences in concentration between the studied groups was nickel (Ni). The level of nickel in the obese women with PCOS (BMI ≥ 30) was significantly higher than in nonobese women (BMI < 30). The content of other trace elements in erythrocytes did not differ significantly between the studied groups. Several significant correlations were found within each of the studied PCOS groups: in the group of obese women, the content of zinc (Zn) in erythrocytes positively correlated with prolactin, the content of magnesium (Mg) positively correlated with testosterone, and the content of manganese (Mn) negatively correlated with thyroid-stimulating hormone. In the group of nonobese women, Zn content correlated positively with testosterone, Ni with luteinizing hormone (LH) and estradiol, and Mg negatively correlated with estradiol. Conclusions: The relationship between the level of trace elements and the level of hormones suggests that, in obese women with PCOS, nickel may play a role in inhibiting the processes of folliculogenesis and ovulation. Research on trace elements and their relationship to ovulatory cycles and the development of PCOS may contribute to reducing the consequences of PCOS and, therefore, should be extended.

## 1. Introduction

Polycystic ovary syndrome (PCOS) is the most common endocrinopathy (a disorder of hormone secretion) in women. Depending on the diagnostic criteria, PCOS affects 6% to 21% of premenopausal women (usually ages 18 to 44 years) [1,2]. Although the diagnostic criteria for PCOS only include hyperandrogenism, oligo/anovulation, and polycystic ovarian morphology [3], many women with PCOS (30–75%) are also overweight or obese. Adipose tissue is an endocrine organ affecting the action of many hormones. Hormonal disorders may contribute to the metabolic disorders accompanying obesity [4,5]. A two-way relationship exists between obesity and PCOS: they both exacerbate each other in an endless, cyclical manner [6]. In overweight and obese women, androgen levels are higher, contributing to hirsutism and excess seborrhea [3,7], fertility is considerably reduced, and miscarriage rates are significantly elevated. Spontaneous miscarriages are higher than in women with normal body weight [8,9]. Obesity may disturb ovulation, lead to a decrease in FSH and luteinizing hormone (LH) secretion, and interfere with the secretion of progesterone [10,11]. Obesity is a major determinant of many long-term consequences of PCOS, including glucose intolerance and the risk of cardiovascular disease [8]. Obesity and mineral disorders, as mentioned by Kurdoglu et al. [12], may contribute to endocrine disruption.

Of the human body, 3% is trace elements. The mineral homeostasis of the body is tightly regulated, and deficiencies in essential minerals can, in turn, affect a wide range of physiological processes. The role of trace elements in the formation of polycystic ovary syndrome is almost unknown, although trace elements have been proven to have a strong influence on the normal metabolism of the body through interaction with many enzymes and hormones. Trace elements, including zinc, play an important role in the proper course of ovulation [13]. Disturbances in the concentration of trace elements in the body may contribute to increases in body mass index (BMI) and waist circumference and may cause endocrine disorders [14]. Recent studies indicated that these metabolic effects are related to oxidative damage mechanisms.

Endocrine activity in trace elements is associated with oxidative stress. Toxic elements produce oxygen free radicals (ROS) and reactive forms of nitrogen. These radicals damage lipids, proteins, DNA, and cell membranes and activate apoptosis and tissue degradation [15]. Chen et al. reported that some trace elements catalyze oxidative stress reactions, leading to the formation of oxygen free radicals (ROS) and that ROS reduce insulin promoter activity by pancreatic β cells expressing insulin mRNA [16,17]. There are similar accompanying processes in PCOS. However, it is still unclear whether this process is the cause or the effect. In recent years, more studies have found that insulin resistance (IR) and hyperandrogenemia occur due to oxidative damage in PCOS [18].

Considering the limited amount of data on the influence of trace elements on PCOS, one of our objectives in this study was to determine whether the levels of the analyzed trace elements in the erythrocytes of patients with PCOS depended on the BMI. Another goal was to investigate the correlation between the hormonal profile and the content of trace elements in the erythrocytes of PCOS patients, which may affect the dynamics of hormonal activity and thus the development of metabolic syndrome. We focused on the analysis of erythrocytes, assuming that the level of elements in the serum provides information about the current state of the body but does not describe the reserves accumulated in blood cells.

## 2. Materials and Methods

### 2.1. Study Population

In this study, we included 47 women aged 26 ± 4.7 years (18–38 years) with an average BMI of 30.4 ± 6.3 kg/m^2^ who were diagnosed with PCOS. The diagnosis of PCOS was based on the presence of two of the three Rotterdam criteria [19]: chronic anovulation/oligomenorrhea, hyperandrogenemia (biochemically), and more than 12 follicles on ultrasound. Ovarian examination was performed using an Ultrasound Voluson 730 (GE Health care, Switzerland).

To compare the content of trace elements in erythrocytes in women with PCOS with women without PCOS, the control group (CG) consisted of 16 healthy women (26.7 ± 4.7 years) with a BMI of 23.6 ± 0.9 kg/m^2^ (Table 1). The control group was recruited randomly from healthy women from the local community. Information about the research was advertised on social media. Willingness to participate was reported by phone. During telephone calls, attention was drawn to the lack of changes in eating habits in the 3 months preceding the study (including elimination diets), no chronic diseases (including obesity and gastrointestinal disorders), and no allergies. A total of 27 women agreed to perform the tests, but 11 of them dropped out. The control group was characterized by normal, regular menstrual cycles, and no clinical symptoms of hirsutism. In the control group, an ultrasound was performed to rule out PCOS. Hypertension, ischemic heart disease, diabetes mellitus, and supplementation constituted the exclusion criteria in both groups.

The characteristics of the respondents are presented in Table 1 and Table 2. None of these women had ever been professionally exposed to heavy metals or trace elements. In both PCOS groups, all women had reduced physical activity of less than 3 h per week. The control group corresponded in this respect to the test group.

Knowing that the dietary intake of trace elements influences their concentration in the body, the diets of PCOS and healthy patients were analyzed. All surveyed women were asked to keep food diaries. The eating habits were analyzed on the basis of 4 days (two weekdays and two weekend days). The contents of selected micronutrients and macronutrients in the diet were calculated using the Diet 6.0 nutritional program recommended by the Food and Nutrition Institute. The diets did not differ statistically in terms of the content of trace elements; therefore, this aspect was omitted in our research. The examined women did not take any supplements and did not smoke cigarettes. In Poland, smoking among women is not popular.

### 2.2. Anthropometric and Biochemical Assessments

Height was measured to an accuracy of 1 cm without standing shoes using a portable height meter. Body weight was measured with an electronic scale with an accuracy of 0.1 kg. Body mass index (BMI) was calculated as the ratio of body weight in kilograms (kg) to the square of height in meters (m^2^). Women were categorized into BMI groups for normal/overweight (18.5–29.9 kg/m^2^) and obese (BMI ≥ 30 kg/m^2^) [20]. The waist-to-hip ratio (WHR) was calculated; for this purpose, we measured the waist circumference (in the narrowest part between the last rib and the pelvic crest at the end of exhalation) and the hip circumference in the widest part by anthropometric measuring tape (±0.5 cm). Total body adiposity was measured under standardized conditions by tetrapolar bioelectrical impedance analysis using a BIA-101 (Akern, Florence, Italy). We measured total body water (TBW), extracellular water (ECW), intracellular water (ICW), fat mass (FM), and muscle mass (MM). The body composition is shown in Table 1.

The biochemical parameter compounds were analyzed as follows: testosterone, insulin and sex hormone binding globulin (SHBG)-electrochemiluminescence (ECLIA), and androstenedione were determined by ELISA (Kobas Rosch E411). The free androgen index (FAI) was calculated using the formula: FAI = 100% × (total testosterone nmol/L/SHBG nmol/L) [21].

Glucose was analyzed by the enzymatic method with the use of hexokinase; fasting glucose and insulin levels were measured; and an oral glucose tolerance test (OGTT, 75 g) was performed. The test was performed on an empty stomach, then the women drank a drink containing 75 g of glucose. After 120 min, the glucose level was measured [22,23]. No women were taking metformin or insulin. The HOMA-IR value was determined according to the formula: HOMA-IR = (fasting insulin mU/mL × fasting glucose mg/dL)/405 [24].

The lipid profile was assessed by the enzymatic method with esterase and cholesterol oxidase. Patients were tested for follicle stimulating hormone (FSH), luteinizing hormone (LH), dehydroepiandrosterone sulfate (DHEAS), and androstenedione (Table 2). Patients with PCOS were diagnosed between the 2nd and 5th day of the cycle. A history was taken from all patients, and a physical examination and a hirsutism assessment were performed according to the Ferriman–Gallwey scale [25].

### 2.3. Sample Analysis

Biochemical analyses were performed in the University Hospital’s Laboratory. Blood samples of 5 mL were collected from fasting women into tubes containing ethylenediaminetetraacetic acid (EDTA). The blood tubes were placed in the refrigerator immediately after collection. To separate the morphotic elements from the plasma, the samples were centrifuged at 3500 rpm for 10 min at 4 °C within 15 min of collection. To ensure the highest quality of research, the blood test tubes used were made of high-purity polypropylene, produced without plasticizers and biocides. Tubes had a Safe-Lock closure, preventing accidental opening of the tube and loss of sample (Eppendorf, Germany). The erythrocyte samples were then immediately frozen at −80 °C and stored as such until the day of analysis.

### 2.4. Chemical Analysis

To ensure the highest quality of research, all reagents used for analyses were of analytical purity. Standard solutions were prepared according to their recipes. For ICP-AES validation, we used working standard solutions of the tested micro- and macroelements (Zn, Ni, Fe, Mn, Cu, and Mg) (Merck KGaA, Germany). All vessels, such as plastic bottles and glass vessels and vials, were rinsed with 15% HNO_3_ and double distilled water (Barnstead, Easypure UV) and dried. Prior to analysis, the erythrocyte samples were mineralized in an MDS-2000 microwave oven by adding 5 mL of nitric acid (69% Merck KGaA, Darmstadt, Germany) and 2 mL of H_2_O_2_ (35% *w/v*) to 1 mL of erythrocytes. In addition to the erythrocyte samples, blank samples were simultaneously mineralized using the same procedure. The samples were mineralized in polytetrafluoroethylene (PTFE) tubes. The following oven parameters were used: maximum power of 1000 W, maximum pressure of 13.8 bar, and maximum operating temperature of 200 °C. The samples were stored in LDPE bottles (Hünersdorff GmbH, Ludwigsburg, Germany) until analysis at 4 °C.

The contents of trace elements in the samples were determined using an ICP-AES apparatus (Yobin Yvon JY-24) with a Meinhard TR 50-C1 nebulizer. Generator parameters were 1000 W output power and frequency 40.68 MHz, and the plasma gas, the auxiliary gas, and the nebulizer gas were argon with flow rates of 12.0, 1.0, and 1.1 mL/min, respectively. The carrier gas flow rate was optimized for the maximum signal-to-background ratio. The following wavelengths were used: Zn, 213.9 nm; Ni, 231.6 nm; Fe, 238.2 nm; Mn, 257.6 nm; Cu, 237.4 nm; Mg, 279.5 nm. All samples were analyzed in triplicate analytical duplicates. The accuracy and precision of the methods used were verified with certified reference material Trace Elements Whole Blood L-1 (Seronorm, Norway). The linearity of the method was verified by creating calibration curves for each element by plotting the peak area of the optimal emission line from the concentration of the standard solutions. We assessed the slope, intersection, and coefficient of determination by least squares linear regression analysis. The accuracy of the method was assessed using the deviation between the nominal and measured concentrations of the spiked samples, known as relative recovery (R%), and the precision of the method was evaluated using relative standard deviation (RSD%) by performing five replicates of the sample solutions. The recoveries of the examined elements (Zn, Ni, Fe, Mn, Cu, and Mg) were 98.5%, 97.4, 97.6%, 99.2%, 98.4%, and 98.6%, respectively. Ten separate blank solutions were prepared and independently analyzed to determine the limits of detection (LODs) and limits of quantification (LOQs) [26]. The LODs and LOQs of the analyzed trace elements were calculated from the standard deviations (SDs) of 10 measurements of blank samples: LOD = 3 × SD and LOQ = 10 × SD. The LOD and LOQ were, respectively, as follows (µg/L): Zn (1.2, 3.37); Ni (0.7, 2.5), Fe (0.85, 2.7); Mn (0.6, 2.0); Cu (1.2, 3.6); Mg (0.6, 2.0). To check for possible contamination during the digestion and sample handling procedure, a blank solution was prepared and passed through the instrument preparation and labeling process with each of the 10 samples.

### 2.5. Statistical Analysis

Statistical calculations were performed using the Statistica software package, version 13.3 (Polish version: StatSoft, Krakow, Poland). Data are expressed as the mean with standard deviation (mean ± SD). Pearson’s correlation statistics were used to investigate the correlation between the variables. The hormonal profile and the content of elements in erythrocytes were compared in nonobese and obese women with PCOS. The differences between the groups were tested using Student’s *t*-test. In the absence of a normal distribution and homogeneity of variance, the differences between the groups were analyzed using the nonparametric Mann–Whitney U test. Measures of variables between more than two groups were compared using ANOVA. To ensure that the analyses performed were correct, Tukey’s post hoc reasonable significant difference (RIR) test was performed, which confirmed the correctness of the analyses. The power of tests that showed statistical significance was above the recommended level of 0.9.

## 3. Results

We found no statistically significant differences between the PCOS group and the control group in age, height, TBW IN and TBW EX, BCM, or WHR (Table 1).

The WHR and BMI values differed significantly between the analyzed groups (Table 1). The remaining anthropometric parameters of the studied patients differed statistically and were significantly higher in obese women with PCOS. These women also differed in biochemical parameters, especially HDL and LDL levels, glucose test after 2 h, and insulin test after 2 h. Women with a BMI ≥ 30 had higher glucose and insulin levels 120 min after the oral administration of 75 g glucose, as well as LDL and HDL values, than women with BMI < 30 (Table 2).

The comparison of trace element levels in red blood cells between obese and nonobese PCOS patients is presented in Table 3. We found no differences in the concentrations of Zn, Fe, Mn, Cu, and Mg between the two PCOS groups and the control group (Table 3). The concentrations of all analyzed elements did not differ significantly between the groups of PCOS women except for nickel.

Table 4 and Table 5 show the significant correlations between hormonal parameters and the contents of trace elements in PCOS erythrocytes. In the group of nonobese women, we found positive correlations between testosterone and zinc levels; estradiol and nickel; LH and nickel; and androstenedione and manganese; and one negative correlation: estradiol and magnesium.

In the group of obese women, a positive correlation was observed between prolactin and zinc as well as testosterone and magnesium, and a negative correlation was observed between TSH and manganese.

## 4. Discussion

### 4.1. Differences in Hormonal Profile between Obese and Nonobese Women with PCOS

In our research, in agreement with Moran et al. [27], we found no differences in the DHEAS levels in women with PCOS with different BMIs. DHEA appears to have a positive effect on antral follicle number and ovarian volume in women undergoing assisted reproduction for primary failure [28]. Although approximately 40–70% of women with PCOS have elevated levels of DHEA-S, the correlation between DHEA-S level and the biochemical parameters of PCOS has not been thoroughly investigated [5,29].

In this study, we found a slightly lower amount of androgens in obese women (BMI ≥ 30), but the difference was not statistically significant. Dunaif et al. [30], in agreement with our results, reported a lower level of androstenedione in obese patients than in nonobese patients. Lerchbaum et al. [31] found no significant differences in the concentration of androstenedione between obese and nonobese women with PCOS. The next parameters we examined were hormones such as estradiol, prolactin, TSH, LH, and FSH. In this study, estradiol levels in obese women with PCOS were significantly higher than in nonobese women. Other authors stated similarly [32,33]. Lerchbaum et al. [31] found, however, that estradiol did not differ significantly between obese and nonobese PCOS groups. The most important function of estradiol in a woman’s body is to control the proper development of the organs of the reproductive system and their structure; estradiol also regulates the menstrual cycle and affects women’ fertility. The maturation of the ovarian follicles also depends on the concentration of estradiol [34]. The level of prolactin did not differ significantly between obese and nonobese women with PCOS. Knowing that prolactin is involved in increasing the number of fat cells and that its increased level increases the secretion of leptin (the satiety hormone), it is suspected that the levels of this hormone would be significantly different between obese and nonobese women [35,36]. In this study, LH and FSH levels in obese and nonobese women with PCOS were similar. The same results were obtained by Saaida [10]. We did not observe any significant correlations between FSH levels and the selected trace elements. We also did not find any literature that indicated the existence of such relationships.

### 4.2. Relationships between Trace Elements and Hormonal Profile in Obese and Nonobese Women with PCOS

Many trace elements are essential for the proper functioning of many biochemical reactions, especially as enzyme cofactors. This mainly applies to processes related to the regulation of glucose homeostasis, either in glucose metabolism or in the control of hormones, especially of insulin. The role and importance of trace elements such as zinc, nickel, iron, manganese, copper, and magnesium in the reactions of PCOS are not fully understood. This study was conducted to verify whether relationships exist between trace elements and hormone balance in women with PCOS and whether the occurrence of obesity changes these relationships [12,13,14,15]. The level of zinc in erythrocytes was comparable in all analyzed groups (PCOS divided into BMI and control group). Our findings are consistent with those of Guler et al. [37]; however, the levels were analyzed in various biological materials. Some researchers found a decrease in serum zinc concentration in women with polycystic ovary syndrome [38,39]. Other authors, such as Chakraborty et al. [39] and Kurdoglu et al. [12], observed an increased level of this element in women with PCOS. However, the aforementioned authors studied the levels of the elements in the blood serum. Analyzing the hormonal profile of women with PCOS, we found a positive correlation between the contents of zinc and prolactin in obese women. Tatarchuk et al. [40] found a similar relationship. Prolactin releases dopamine, which is the main neurotransmitter in the hypothalamus, where it controls the production of many hormones. Dopamine and prolactin form a feedback loop [41,42]. The regulation of prolactin is also influenced by estrogens, which increase the production and secretion of prolactin from the pituitary gland. Such a relationship leads to an increase in the amount of prolactin and the inhibition of the secretion of gonadotropins, which are responsible for the maturation of Graff’s follicles and ovulation [42,43,44]. We observed a positive correlation between nickel and estradiol in nonobese women with PCOS. Tatarchuk et al. [40] and Spritzer et al. [45] found that elevated estradiol levels increased the amount of nickel in red blood cells (*p* < 0.05), whereas Zheng et al. [37] found opposite relationships in blood serum. Nickel is classified by the WHO as a group of potentially essential micronutrients, but literature data regarding its role in regulating reproductive function are virtually absent. Due to the ability of Ni to change the activity of macrophages, inhibit natural killers, and induce inflammation, this trace element can promote inflammation and protection of the reproductive system in this category of women [27,46,47]. Nickel has a serious adverse effect on the hypothalamic–pituitary–gonadal axis [47]. While analyzing the hormonal profile, we observed a significant positive correlation between nickel and LH in nonobese women, despite the lack of significant differences in nickel concentration in erythrocytes between obese and nonobese women. However, in other studies [38], no similar relationship was found. In this study, no significant correlation was found between iron parameters and the hormonal profile in the PCOS groups, which would explain the role of obesity. This result was expected because adipose tissue has a mild effect on iron stores, which are found mainly in the liver. Obesity may promote iron deficiency by inhibiting the absorption of iron from the duodenum in the diet [8,45,48]. Statistical analysis showed no significant correlation between the level of this element in erythrocytes and the hormonal profile of women with PCOS. Repetition of the study on an even larger group of women with PCOS from different ethnicities and regions of residence is required. Analyzing the relationship between the hormonal profile and trace elements, we observed that with the increase in manganese concentration in nonobese women, the level of androstenedione increased. The effect of obesity on blood androgen production in PCOS has not yet been clearly established [27]. Manganese is an element that participates in the mechanisms that protect the body against oxidative damage. Manganese reduces the high reactivity of superoxide ions to less reactive hydrogen peroxide compounds [40]. When looking for the association of trace elements with TSH, we found only one negative correlation between TSH and manganese in obese women. Nonobese women did not show this relationship. When examining the hormonal profile of PCOS patients, similar to Khalaf et al. [49], we did not notice significant dependences related to the level of copper in erythrocytes in obese and lean women with PCOS. Other authors [5,38] observed that serum Cu concentration was higher in PCOS patients than in the control group. In addition, they found that serum LH and testosterone levels increased with Cu levels. Many authors argued that a link exists between Cu and PCOS through the effects of reactive oxygen species (ROS) on hormone levels [50,51]. Our research showed that in women with PCOS, regardless of the presence or absence of obesity, the concentration of magnesium in erythrocytes was comparable with that in the control group. Similar results were obtained by Tatarchuk et al. [40] in a study of the blood serum of women with PCOS and a control group. There are literature data showing lower serum magnesium levels in obese people without PCOS than in people with normal body weight [52,53,54]. In this study, we observed a positive correlation between the levels of magnesium and testosterone in erythrocytes in obese women. Unfortunately, no similar results were found in the available literature on erythrocytes in the blood serum of women with PCOS [55]. Analyzing the hormonal profile of women with PCOS, a negative correlation between Mg and estradiol was found in nonobese women. Magnesium supports the detoxification of estrogens by directly increasing the activity of glucuronyl transferase [56]. Ovarian hormones affect magnesium levels by causing magnesium levels to drop at certain times in the menstrual cycle and by changing the calcium–magnesium ratio. These cyclical changes can cause many of the well-known symptoms of premenstrual syndrome (PMS) in women with magnesium and/or calcium deficiency [50]. The results of our research, similar to Tatarchuk et al. [40] and Babapour et al. [57], seem to confirm the negligible influence of magnesium on the course of PCOS.

## 5. Conclusions

The metabolism of trace elements may be impaired in obese patients; for some, the exact frequency of this change is still unknown. The identified increase in the level of nickel in erythrocytes in obese women with PCOS may indicate its potential role in inhibiting the processes of folliculogenesis and ovulation. Therefore, monitoring minerals in the body and their consumption may be recommended in obese women with PCOS. It is also suggested that monitoring minerals in obese women in serum may not be the best option in assessing body supply.

## Figures and Tables

**Table 1:** Characteristics of patients with PCOS.

Parameters	Nonobese (PCOS with BMI < 30) *n* = 23		Obese (PCOS with BMI ≥ 30) *n* = 24		Control Group (CG) *n* = 16		*p*-Value		
	Mean	SD	Mean	SD	Mean	SD	BMI < 30 vs. BMI ≥ 30	BMI ≥ 30 vs. CG	BMI < 30 vs. CG
Body height (m)	1.67	0.06	1.65	0.06	1.66	0.06	0.31118	0.88195	0.60858
Body weight (kg)	70.3	8.2	96.1	13.3	**65.06**	**6.28**	**0.00012**	**0.00012**	0.24821
BM (kcal)	1421.7	82.2	1612.6	179.0	**1407.2**	**98.3**	**0.00020**	**0.00018**	0.93642
TM (kcal)	2061.1	134.5	2274.7	237.9	**2040.6**	**82.3**	**0.00084**	**0.00056**	0.92731
TBW (L)	33.6	3.3	39.9	4.6	32.1	2.4	**0.00013**	**0.00012**	0.41378
TBW IN (L)	18.9	8.1	22.0	4.3	17.1	1.6	0.21225	**0.03559**	0.59377
TBW EX (L)	16.4	1.9	17.9	3.2	15.6	1.8	0.13149	**0.01841**	0.58027
Fat mass (%)	35.3	8.3	42.9	5.6	**27.2**	**2.9**	**0.00157**	**0.00012**	**0.00100**
Fat mass (kg)	24.5	5.7	41.6	9.7	**20.3**	**3.5**	**0.00012**	**0.00012**	0.17126
BCM (kg)	23.2	2.8	29.1	7.8	**23.1**	**3.9**	**0.00349**	**0.00544**	0.99969
BCM (%)	50.5	3.3	54.7	7.6	**51.1**	**3.3**	**0.03979**	0.12107	0.92069
Muscle mass (kg)	32.2	7.7	38.1	9.0	**30.9**	**4.6**	0.05220	**0.02123**	0.86335
Muscle mass (%)	45.68	11.05	39.19	9.04	47.7	3.6	0.07270	**0.02000**	0.77245
Waist circumference (cm)	88.53	8.86	112.88	10.80	**74.0**	**2.56**	**0.00010**	**0.00012**	**0.00013**
Hip circumference (cm)	103.10	6.27	116.03	7.96	**95.06**	**2.59**	**0.00012**	**0.00012**	**0.00087**
BMI (kg/m^2^)	**25.2**	**2.8**	**35.3**	**4.4**	**23.57**	**0.9**	**0.00102**	**0.00102**	**0.00183**
WHR	0.86	0.05	0.97	0.06	**0.80**	**0.020**	**0.00012**	**0.00012**	**0.00068**

Significance differences (*p*-value < 0.05) are marked in bold; BM, basic metabolism; TM, total metabolism; TBW, total body water; TBW IN, total intracellular water; TBW EX, total extracellular water; BCM, body cell mass; BMI, body mass index; WHR, waist–hip ratio.

**Table 2:** Biochemical differences between nonobese (BMI < 30) and obese (BMI ≥ 30) PCOS patients.

PCOS	Nonobese (PCOS with BM 0) *n* = 23		Obese (PCOS with BMI ≥ 30) *n* = 24		Total PCOS Patients *n* = 47		*p*-Value
	Mean	SD	Mean	SD	Mean	SD	BMI < 30 vs. BMI ≥ 30
DHEA-SO_4_ (μg/d)	248.6	74.2	250.6	102.6	249.5	87.1	0.94763
Androstenedione (ng/mL)	3.94	1.69	3.56	1.30	3.77	1.52	0.47922
TSH (mIU/mL)	1.77	0.53	1.75	0.39	1.76	0.46	0.88547
LH (mIU/mL)	7.47	1.81	7.26	2.71	7.37	2.24	0.79246
FSH (mIU/mL)	5.34	1.10	5.24	1.06	5.29	1.07	0.78837
Estradiol (pg/mL)	**37.93**	**28.81**	**49.81**	**9.86**	43.39	22.74	**0.02253**
SHBG (nmol/L)	39.25	17.53	29.80	10.53	34.79	15.22	0.06900
Testosterone (ng/mL)	0.544	0.234	0.598	0.198	0.569	0.217	0.46825
FAI (%) Prolactin (ng/mL)	1.38 18.12	1.11 5.02	2.01 16.49	0.89 6.81	1.64 17.37	1.03 5.88	0.14834 0.42696
Insulin test 0 (mU/L)	10.42	13.00	17.14	5.17	13.51	10.61	0.06287
Insulin test after 2 h (mU/L)	**54.30**	**37.82**	**118.14**	**87.54**	83.63	72.12	**0.00750**
Glucose test 0 (mg/dL)	88.7	9.3	94.0	11.6	91.1	10.6	0.14817
Glucose test after 2 h (mg/L)	**105.8**	**24.3**	**129.9**	**33.3**	116.8	30.9	**0.01969**
HOMA-IR (mg/dL) Total cholesterol (mg/dL)	**2.26** 188.6	**1.61** 23.8	**3.98** 185.4	**2.02** 29.5	3.04 187.1	1.78 26.4	**0.04571** 0.73124
LDL (mg/dL)	**105.9**	**19.7**	**126.1**	**29.7**	115.7	26.7	**0.02440**
HDL (mg/dL)	**65.63**	**19.81**	**48.23**	**12.91**	57.18	18.77	**0.00504**
TG (mg/dL)	104.0	54.5	129.6	77.7	116.4	67.0	0.271147

Significance differences (*p*-value < 0.05) are marked in bold: DHEA-SO_4_, dehydroepiandrosterone sulfate; TS, thyroid-stimulating hormone; LH, luteinizing hormone; FSH, follicle-stimulating hormone; SHBG, sex hormone-binding globulin; FAI, free androgen index; HOMA-IR, homeostatic model assessment, insulin resistance; LDL, low-density lipoprotein; HDL, high-density lipoprotein; TG, triglyceride.

**Table 3:** Comparison of mineral content in erythrocytes (µg/mL) between the groups of obese and nonobese women with PCOS and the control group.

Minerals (µg/mL)	Non-Obese (PCOS with BMI < 30) *n* = 23		Obese (PCOS with BMI ≥ 30) *n* = 24		Control Group (CG) *n* = 16		*p*-Value	*p*-Value	*p*-Value
	Mean	SD	Mean	SD	Mean	SD	BMI < 30 vs. CG	BMI ≥ 30 vs. CG	BMI < 30 vs. BMI ≥ 30
	(range)		(range)		(range)				
Zn	10.62	1.40	10.35	1.29	10.50	1.34	0.94965	0.98104	0.83979
	(8.49–14.47)		(8.056–12.9)		(8.47–12.99)				
Ni	0.001	0.001	**0.002**	**0.001**	0.001	0.001	**0.00512**	0.69120	**0.01782**
	(<LOD–0.002)		(<LOD–0.003)		(<LOD–0.002)				
Fe	861.0	164.9	910.4	56.4	845.8	84.9	0.94258	0.35126	0.44680
	(545.8–1333.7)		(812.0–1022.3)		(718.7–978.7)				
Mn	0.019	0.006	0.017	0.004	0.021	0.007	0.75677	0.31455	0.66500
	(<LOD–0.029)		(0.011–0.023)		(0.015–0.036)				
Cu	0.73	0.13	0.71	0.07	0.78	0.14	0.53749	0.28812	0.86097
	(0.33–0.94)		(0.632–0.847)		(0.57–1.10)				
Mg	49.86	8.15	51.63	5.99	50.68	7.20	0.65630	0.30992	0.77232
	(31.22–66.82)		(42.49–64.64)		(28.44–56.81)				

Significant differences (*p*-value < 0.05) are marked in bold.

**Table 4:** Correlation coefficients between the level of trace elements (µg/mL) and the biochemical parameters in nonobese women with PCOS (BMI **<** 30).

Parameter	Zn	Ni	Fe	Mn	Cu	Mg
DHEA-SO_4_ (μg/d)	−0.020	0.028	−0.338	0.456	0.265	−0.189
Androstenedione (ng/mL)	0.021	0.285	0.016	**0.513**	−0.188	0.162
TSH (mIU/mL)	−0.069	0.361	0.237	0.150	0.288	0.289
LH (mIU/mL)	0.034	**0.587**	0.325	0.113	−0.244	0.419
FSH (mIU/mL)	−0.143	0.248	0.142	−0.023	−0.170	0.459
Estradiol (pg/mL)	−0.300	**0.506**	−0.313	−0.005	−0.267	**−0.580**
SHBG (nmol/L)	0.291	−0.131	0.345	−0.418	−0.080	0.035
Testosterone (pg/mL) FAI	**0.504** −0.197	0.340 0.034	0.365 −0.240	0.309 0.413	0.189 −0.059	−0.014 −0.292
Prolactin (pg/mL)	0.133	−0.240	−0.445	−0.204	0.241	−0.181

The correlations with *p*-values greater than 0.05 are marked in bold: DHEA-SO_4_, dehydroepiandrosterone sulfate; TSH, thyroid-stimulating hormone; LH, luteinizing hormone; FSH, follicle-stimulating hormone; SHBG, sex hormone-binding globulin; FAI, free androgen index.

**Table 5:** Correlation coefficients between the level of trace elements (µg/mL) and the biochemical parameters in obese women with PCOS (BMI ≥ 30).

Parameter	Zn	Ni	Fe	Mn	Cu	Mg
DHEA-SO_4_ (μg/d)	−0.018	−0.287	0.357	−0.239	−0.322	−0.062
Androstenedione (ng/mL)	−0.045	−0.166	0.062	−0.266	−0.369	0.243
TSH (mIU/mL)	−0.420	−0.454	−0.012	**−0.517**	−0.270	−0.067
LH (mIU/mL)	−0.147	0.048	0.294	−0.318	0.038	0.064
FSH (mIU/mL)	−0.478	0.112	−0.104	−0.421	−0.413	−0.242
Estradiol (pg/mL)	0.078	−0.208	0.233	0.224	−0.050	−0.093
SHBG (nmol/L)	−0.207	−0.022	−0.188	0.179	0.058	−0.028
Testosterone (pg/mL) FAI	−0.145 0.244	−0.034 0.111	0.097 0.370	−0.059 −0.063	−0.023 −0.106	**0.593** 0.360
Prolactin (pg/mL)	**0.613**	0.174	0.071	0.298	0.128	0.266

The correlations with *p*-values greater than 0.05 are marked in bold: DHEA-SO_4_, dehydroepiandrosterone sulfate; TSH, thyroid-stimulating hormone; LH, luteinizing hormone; FSH, follicle-stimulating hormone; SHBG, sex hormone-binding globulin; FAI, free androgen index.

## Data Availability

The data presented in this study are available on request from the corresponding author.
